# An adaptive simulation intervention decreases emergency physician physiologic stress while caring for patients during COVID-19: A randomized clinical trial

**DOI:** 10.1371/journal.pone.0331488

**Published:** 2025-09-03

**Authors:** Leigh V. Evans, James W. Bonz, Samuel Buck, Jeffrey N. Gerwin, Shacelles Bonner, Suzette Ikejiani, Tatiana Moylan, Melissa Joseph, Gustavo de Oliveira Almeida, Jessica M. Ray, James D. Dziura, Arjun K. Venkatesh, Ambrose H. Wong

**Affiliations:** 1 Department of Emergency Medicine, Yale School of Medicine, New Haven, Connecticut, United States of America; 2 Department of Emergency Medicine, NYU Grossman School of Medicine, New York, New York, United States of America; 3 Department of Emergency Medicine, The Johns Hopkins University School of Medicine, Baltimore, Maryland, United States of America; 4 Langley Memorial Hospital, Langley, British Columbia, Canada; 5 University of British Columbia, Vancouver, British Columbia, Canada,; 6 University of Arizona Health Sciences, Phoenix, Arizona, United States of America; 7 Department of Health Outcomes & Biomedical Informatics, University of Florida College of Medicine, Gainesville, Florida, United States of America; 8 Office of Quality and Patient Safety, St. Jude Children’s Research Hospital, Memphis, Tennessee, United States of America,; 9 Yale School of Public Health, New Haven, Connecticut, United States of America,; 10 Department of Population Health, NYU Grossman School of Medicine, New York, New York, United States of America; Kirklareli Universitesi, TÜRKIYE

## Abstract

**Background:**

Stressful work environments and burnout in emergency medicine (EM) physicians adversely impact patient care quality. The future EM workforce will need to prioritize clinician well-being to ensure optimal patient care.

**Methods:**

This prospective, randomized, controlled study aimed to determine whether an adaptive simulation intervention, COVID-19 Responsive Intervention: Systems Improvement Simulations (CRI:SIS), decreased physiologic stress as measured by heart rate variability (HRV) in front-line EM physicians during the COVID-19 pandemic. HRV was measured with smart shirts and self-reported State-Trait Anxiety Inventory (STAI) were collected at baseline and during four 8-hour clinical shifts for all participants. The intervention group (n = 40) received a 3-hour virtual educational simulation intervention consisting of four simulation scenarios (CRI:SIS). The control group (n = 41) received no simulation intervention.

**Results:**

There were no significant differences in demographics between groups. HRV data collected from 81 physicians across a total of 324 clinical shifts showed an increase in HRV (decrease in physiologic stress) in shifts immediately following CRI:SIS in the intervention group as measured by a root mean square standard deviation (RMSSD) difference of 11.55 ms (95% CI, −19.90 to −3.20; *P* = 0.007) compared to the control group. Post-intervention STAI did not significantly differ between intervention and control.

**Conclusion:**

An adaptive simulation-based educational intervention led to decreased physiologic stress (increased HRV) among emergency physicians who received a simulation education intervention. Reduced physiologic stress generated by adaptive simulation interventions may improve both patient safety and clinician well-being.

## Introduction

The extensive and long duration of response to the coronavirus 2019 (COVID-19) pandemic increased clinician work burden while creating an environment of uncertainty, raising concerns about increasing burnout and attrition of front-line health workers who were directly diagnosing and managing critically ill patients. [1–4] The relationship between COVID-19-related stress and work intentions showed that approximately 1 in 3 physicians and nurses surveyed intended to reduce work hours. One in 5 physicians and 2 in 5 nurses intended to leave their practice altogether. [5] Of those surveyed, 74.7% of emergency physicians reporting burnout symptoms since the start of the pandemic. [6] Reducing burnout and improving a sense of feeling valued may allow healthcare organizations to better maintain their workforce post-pandemic. [7–9] These work trends are costly; turnover and reduced clinical hours due to physician burnout in the United States cost $4.6 billion annually. [10] Physician burnout is also linked to increased medical errors, increased risk of patient safety incidents, extended patient waiting times, and decreased patient satisfaction. [11–13]

Due to the increasing prevalence of burnout in the healthcare sector, recent research has increasingly focused on methods to measure stress in both simulated and in situ clinical environments. The emergency care workforce has one of the highest rates of burnout and attrition with the highest negative impact from the pandemic. [14] Our recent work outlining the impact of COVID-19 on Emergency Department (ED) clinicians highlights the importance of supporting clinicians along a range of psychological needs. If these needs are unmet, ED healthcare workers may be at an increased risk of infection, anxiety, and burnout. [4,15] Experts have advocated for more accurate and early detection of stress amongst healthcare personnel to develop and intervene prior to development of severe burnout symptoms. [16–20]

During acute stress events, healthcare workers may experience activation of the sympathetic nervous system resulting in key physiological changes. [21] Established markers of this stress response include a decrease in heart rate variability (HRV). [22] Evidence suggests links between physiologic measures of acute stress, particularly HRV, and the emotional exhaustion subscale of burnout. [23,24] HRV is the measure between consecutive heartbeats and is controlled by the autonomic nervous system. Increased parasympathetic activity decreases heart rate via the vagus nerve. Therefore, increased vagal tone leads to increased HRV. An increase in HRV correlates with decreased stress. [25] Low HRV has been observed in individuals presenting with burnout resulting from repeated or continuous stress exposure. [26] For workers presenting with clinical burnout, measures of HRV have been shown to be lower than both workers with non-clinical burnout and healthy individuals with no burnout symptomology. [26] Such low levels of HRV suggest sympathetic predominance which may contribute to the adverse health effects associated with clinical burnout. [23,26,27]

In March of 2020, EDs began seeing increased volumes of COVID-19-positive patients. Rapidly changing hospital protocols, as well as the intense, challenging work of caring for an influx of severely ill patients, created new stressors including the necessity to adopt rapidly changing clinical guidelines. The challenges facing healthcare clinicians were exacerbated by the novelty of COVID-19 and the constantly evolving clinical understanding of the disease. [1,2] Within our own institution, daily and weekly emails from both the hospital and ED administration provided rapidly updating guidance on evolving COVID-19 protocols. These communications occurred as frequently as 200 per month.

### Context and goal of this study

Healthcare simulation, a growing field that uses technology to improve safety and care quality, has shown increasing promise in improving hospital and clinician preparedness during rapidly evolving pandemics like COVID-19. [28–30] However, evaluation of its impact on clinician stress and burnout has thus far been limited. [31] Accordingly, we developed and piloted a new rapidly adaptive simulation intervention to increase guideline adoption and preparedness for COVID-19 care delivery. The objective of this study was to evaluate the effect of simulation–based training and quality improvement intervention (COVID-19 Responsive Intervention: Systems Improvement Simulations – CRI:SIS) on emergency physician physiologic stress. To our knowledge, this was the first clinical trial examining the effect of a simulation intervention on physiologic stress amongst emergency physicians.

## Methods

### Setting and study design

This prospective, randomized controlled trial (RCT) of emergency medicine (EM) resident and faculty physicians occurred at two sites of a teaching hospital system located in an urban city within the Northeast United States: (1) a tertiary care referral center with an average annual adult volume of 100,000 visits; and (2) an urban community hospital with an average annual adult volume of 65,000 visits. Eligible participants were enrolled from January 1 to December 31 2021. This trial period encompassed the second wave, Delta surge, and third wave of the COVID-19 pandemic.

All participants wore Hexoskin Smart Shirts (Carré Technologies Inc.) with embedded textile sensors that allow for continuous 1-lead ECG recording to measure HRV. A baseline session established rest measurements, baseline State-Trait Anxiety Inventory assessment, and collection of demographic information as well as potential confounding factors related to HRV measurement (e.g., caffeine intake, Body Mass Index, etc.). The intervention group wore smart shirts during two pre-CRI:SIS and two post-CRI:SIS ED clinical shifts. The control group wore the shirts during four clinical shifts with no simulation intervention. Data collection concluded after all enrolled participants completed the baseline measurement and four data collections. Our institutional review board approved this study and we obtained written consent for each participant. We followed the CONSORT reporting guidelines for randomized control trials. [32]

### Ethics approval

The study protocol was approved by the Yale University Institutional Review Board (HIC# 2000029372). All recruited physicians were fully briefed and given the opportunity to ask questions regarding the details of the study before either agreeing or declining to participate. Each participant submitted signed written statements of informed consent that outlined the study’s objectives, the safety risks of participating, the requirements of the participants, and permission to publish their deidentified biometric data before commencing involvement in the study.

### Participants and recruitment

The target participant demographics were frontline EM physicians, consisting of resident and attending physicians who were actively treating acutely ill COVID-19 patients in the ED at either of the two sites. Physicians were recruited via email or in person. Trial exclusion criteria included the use of beta-blockers and antiarrhythmic medication, active thyroid dysfunction, and pregnancy. We ended recruitment when we reached our target sample size plus an additional two participants per arm to accommodate for potential loss to follow-up.

### Randomization

After completing two clinical shifts, participants were randomized 1:1 to either the control or intervention group using a computer-generated randomization sequence. [33] Permuted block randomization was performed and randomization was stratified by clinical experience level: Junior (PGY 1–3) or Senior (PGY 4, attendings). Randomization was conducted using the sequentially numbered, opaque, sealed envelope (SNOSE) technique prepared by a statistician prior to the start of the clinical trial. A research associate not involved with delivering the intervention opened a corresponding envelope designating the participant to either the control or intervention arm. The randomization list was kept in a secure cloud-based electronic file and the randomization sequence was blinded from investigators and participants. All participants were randomized within 12 hours of completing their baseline data collection.

### Intervention

The intervention group completed a 3-hour virtual training simulation (CRI:SIS), using Laerdal LLEAP simulation software, between 1–10 days prior to their next scheduled clinical shift. The control group wore smart shirts during four clinical shifts and received no simulation intervention. All participants had similar access to COVID-19 Task Force updates, guidelines, weekly town hall meetings, and any in-service support available to clinical staff. After the study had been completed, the control group was offered the simulation intervention for its educational benefit.

The intervention consisted of a carefully tailored group of four intervention scenarios (S1 Table) with varying severity of COVID-19 presentations requiring appropriate communication, clinical management, and disposition of patients. Specific critical actions were adapted over the course of the study as guidelines and recommendations evolved in consultation with administrative leadership. The scenarios were administered by one of two trained co-investigators with expertise in healthcare simulation and board-certified in emergency medicine. The study principal investigator, who was also a board-certified emergency physician and a healthcare simulation expert, was present for every scenario to ensure consistency in the intervention delivery. The scenarios were conducted in the same sequence for every participant and focused on the four following objectives:

1. “CMO” (comfort measures only): Decision-making regarding goals of care and potential withdrawal of care in the ED in a patient presenting with severe illness and poor prognosis with extremely low chances of survival in the next 48 hours
2. Mild COVID-19: Understanding discharge criteria, adapted to address vaccine hesitancy
3. Moderate COVID-19: Familiarity with COVID-19 Severity Index [34] to determine the level of care required, disposition, and potential for clinical deterioration
4. Severe COVID-19: Management of complications associated with severe hypoxemia including ventilator management and treatment of pneumothorax

Each intervention session began with a prebriefing to discuss recent changes in clinical guidelines and was followed by a structured debriefing session led by a member of the research team with clinical expertise in emergency medicine and advanced debriefing skills. Over the course of the study, critical actions were updated, and the scenarios were adapted based on new COVID-19 guidelines at our institution.

### Intervention Adaptations

**Fig 1:**
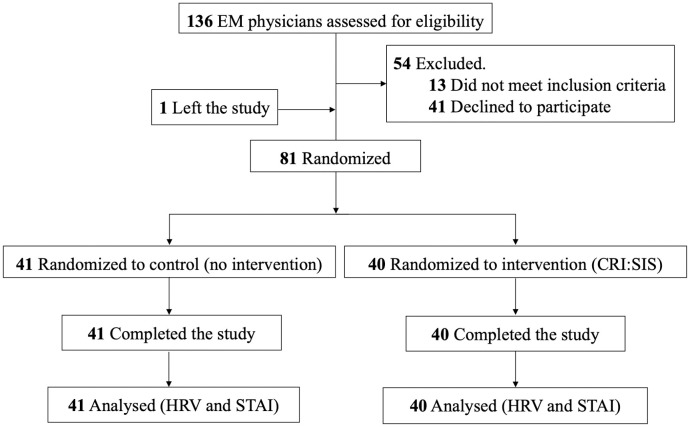
CONSORT flow diagram depicting participant flow through the trial. The diagram outlines the flow of participants through each stage of the randomized controlled trial, from enrollment to analysis. A total of 81 participants enrolled and completed the clinical trial. CONSORT indicates consolidated standards of reporting trials; CRI:SIS, COVID-19 Responsive Intervention: Systems Improvement Simulations.

CRI:SIS successfully made iterative updates throughout the study as guidelines and best practices evolved. The Yale New Haven Hospital (YNHH) COVID-19 Task Force made 149 guideline changes from January through December 2021. We made a total of 22 iterative changes to the intervention over the course of 12 months (30 sessions). S1 Fig 1 shows a timeline of highlighted changes made to our adaptive simulation relative to current events and significant YNHH guideline changes.

### Outcomes measures

Our primary outcomes consisted of physician HRV while on shift. Consistent with prior work on physiologic stress, HRV was assessed as the time-domain measure of root mean square standard deviation (RMSSD) of sequential R-R intervals. [24,35] RMSSD was selected because it was more resistant to respiratory artifacts than other HRV measures, such as the standard deviation of all normal-to-normal intervals (SDNN). [24]

We also included a co-primary outcome of the self-reported State-Trait Anxiety Inventory (STAI). [36,37] STAI is a psychological assessment tool used to measure both state anxiety (how a person feels at a particular moment) and trait anxiety (individual differences in anxiety proneness). It helps to identify and quantify the presence and severity of anxiety symptoms. It can also be useful in monitoring changes in anxiety levels over time and evaluating the effectiveness of treatment interventions. [38] During the baseline session, all participants completed the 40-item self-reported STAI, consisting of 20-item trait anxiety (TA) questions and 20-item state anxiety (SA) questions. A survey including the 20-item SA questions was administered immediately following shift completion. Inventory items were scored on a 4-point ranking scale and summed up to a continuous variable sum score. The survey included a self-reported field indicating the number of resuscitations performed per shift (S2 Table).

We collected basic demographic information from self-reported surveys of participants during the baseline session, consisting of sex, age, race and ethnicity, years of experience in emergency medicine practice at time of enrollment, Body Mass Index, average weekly exercise time in minutes, and average daily caffeine consumption in mg.

### Statistical analysis and sample size calculation

We presented demographic descriptive data by randomized groups as means and standard deviations as appropriate. Analyses of primary outcomes were performed on the intent to treat population using all available outcome data. We used linear repeated measures mixed effects models to compare the primary outcomes of HRV and state anxiety questions of the STAI. A random effect was included for the subject to accommodate correlation between repeated assessments. Models were adjusted for participant activity level (number of steps). Least squares means (LSMeans) were used to describe outcomes pre-intervention (i.e., first two shifts) and post-intervention (i.e., second two shifts). Linear contrasts were used to compare the outcomes between pre-intervention and post-intervention shifts. Comparisons are presented as differences between LSMeans with 95% Confidence Intervals (CI). We used a Bonferroni correction to account for multiple comparisons from the two primary outcomes. All analyses were performed using SAS 9.4 statistical software. All tests were 2-tailed, and P < 0.025 was considered significant.

In order to automate the data derived from wearable sensors, we previously created a tool to both extract meaningful metrics and introduce methodologies for noise filtration and data quality assurance. [39] Using this tool, ECG recordings were algorithmically converted, filtered, and analyzed to calculate RMSSD. Representative 5-minute windows were selected from each recording as this timeframe is considered the conventional recording period to measure short-term HRV. [24] To standardize our window selection for HRV analysis, we selected time windows within a 30-minute period when the participant was involved in a shift sign-out. Our clinical experts determined that end-of-shift sign-out was the optimal choice because it met two essential criteria: 1) participants were not actively exerting themselves and would likely be sitting for at least ten minutes, allowing their HR to return to its resting state, and 2) it was a consistent period at the end of every participant’s shift that would represent cumulative stress. [40] Selected windows met strict quality level criteria (>85% clear R-R intervals) and activity level (<20 steps/5-min) thresholds, captured from the smart garment ECG and accelerometry sensors. Quality thresholds for our raw ECG data were determined by existing guidelines in line with previous literature. [41]

Given an SD of 15ms in HRV, we targeted a sample size of 38 per group providing 80% power at the two-sided 0.025 significance level to detect differences of 10.8ms, an effect reflecting clinically meaningful changes to stress in prior HRV studies. [15,29,30] In addition, a sample size of 38 per group would provide 80% power to detect an estimated STAI score difference of 5.8 with SD = 8 (effect size = 0.72). [42] We planned to include 40 subjects per group to accommodate a potential 5% lost to follow-up.

## Results

A total of 81 participants were enrolled and randomized in the study (mean [SD] age, 35.5 [10] years; 45 [55.6%] women and 36 [44.4%] men). One participant left the study prior to formal initiation of data collection and randomization. None were lost to follow-up. In terms of race and ethnicity, 49 [60.5%] participants identified as Caucasian/White, non-Hispanic; 14 (17.3%) Asian/Pacific Islander; 7 (8.6%) Black or African American, 5 (6.2%) multiple ethnicities or other, 2 (2.5%) Hispanic, and 4 (6.2%) did not disclose their ethnicity. Emergency medicine clinical experience was a mean (SD) of 6 (8) years. A total of 71 (85.2%) participants had a BMI of less than 30, with 11 (14.8%) having a BMI of 30 or greater. The mean physical activity per week was 127.5 (120) minutes. The mean (SD) caffeine intake was approximately 188.5 (122.5) mg per week. At baseline, the control group reported mean (SD) RMSSD of 29.75 (13.26) ms, compared to the intervention group’s 35.13 (23.60) ms. The control group reported a mean (SD) Trait Anxiety (TA) score of 37.90 (10.13) points and a State Anxiety (SA) score of 32.68 (8.31) points, while the intervention group reported a mean (SD) Trait Anxiety (TA) score of 38.83 (11.47) points and a State Anxiety (SA) score of 34.45 (9.83) points. No significant differences were found in any demographic factors, health-related characteristics, and baseline RMSSD and STAI between the control and intervention groups (Table 1). The control group performed a mean (SD) of 2.45 (3.22) resuscitations per shift vs. the intervention group’s 3.22 (7.06) resuscitations per shift with no statistically significant difference between the two groups.

**Table 1:** Demographic information of participating emergency medicine physicians.

	Participants, No. (%)^a,b^	
Characteristic: Measure	Control Group (N = 41)	Intervention Group (N = 40)
**Sex**		
Female	19 (46)	26 (65)
Male	22 (54)	14 (35)
**Age, mean (SD), years**	34 (9)	37 (11)
**Age, range, years**	25, 69	25, 69
**Race/Ethnicity**		
Caucasian/White, non-Hispanic	23 (56)	26 (65)
Black or African American	3 (7)	4 (10)
Asian/Pacific Islander	10 (24)	4 (10)
Hispanic	2 (5)	0
Multiple ethnicites/other	1 (2)	4 (10)
Prefer not to say	2 (5)	2 (5)
**Years of EM experience at time of enrollment**		
Mean (SD)	5 (7)	7 (9)
0-1	8 (20)	10 (25)
2-3	12 (29)	9 (23)
4-5	11 (27)	9 (23)
6-10	2 (5)	1 (3)
10<	8 (20)	11 (28)
**Body Mass Index (BMI)**		
<30	37 (88)	34 (83)
≥30	4	7 (17)
Cannot be determined	1	0
BMI, Range	18.2, 32.6	19.2, 35.5
**Exercise (minutes per week)**		
Mean (SD)	127 (119)	128 (121)
<60	11 (26)	17 (41)
60-120	15 (36)	8 (20)
121-240	9 (21)	10 (24)
240<	7 (17)	6 (15)
**Caffeine (mg)** ^**c**^		
Mean (SD)	191 (122)	186 (123)
0	3 (7)	1 (3)
1-190	19 (46)	22 (55)
191-380	16 (39)	13 (33)
381+	3 (7)	4 (10)

Demographic information of participating emergency medicine physicians, recorded via a self-reported questionnaire at the time of the baseline session (N = 81). Data is presented as No. (%) unless otherwise noted. No statistically significant difference was found between the groups. Abbreviations: EM, emergency medicine; SD, standard deviation.

^a^ Data are presented as number (percentage) of participants unless otherwise indicated.

^b^ Some percentages in this table may not equal 100% due to rounding.

^c^ A standard 8 oz cup of coffee typically contains about 95 mg of caffeine.

Of the 81 study participants, 41 were randomized to the control arm (20 Juniors and 21 Seniors), and 40 were randomized to the intervention arm (20 Juniors and 20 Seniors). Please refer to the CONSORT diagram (Fig 1) for participant flow through the trial.

### Main outcomes

#### Heart rate variability.

The HRV results of 81 participants (41 control and 40 intervention) were evaluated. The post-CRI:SIS clinical shifts were collected on average within three days (range 0–8 days) after the intervention.

Pre-CRI:SIS, the mean RMSSD for the control group was 38.96 ms (SE, 3.96), and the mean RMSSD for the intervention group was 38.29 ms (SE, 4.11). A linear mixed model for repeated measurements, adjusted for activity level (steps), showed that the mean difference in pre-CRI:SIS RMSSD for the intervention group compared to the control group was −0.67 ms (95% CI, −10.678ms to 12.026 ms; *P* = 0.91), indicating no difference in stress between groups. Post-CRI:SIS, the mean RMSSD for the control group was 31.77 ms (2.91), and the mean RMSSD of the intervention group was 43.32 ms (SE, 3.03). In a mixed model, a mean difference in RMSSD for the intervention group compared to the control group was 11.55 ms (95% CI, −19.90 ms to −3.20 ms; *P* = 0.007), showing significantly higher HRV, and therefore lower physiological stress in the intervention group (Table 2) as compared to the control group.

**Table 2:** Comparison of HRV and STAI between intervention and control at Pre-CRI:SIS and Post-CRI-SIS shifts from linear mixed model for repeated measures.

Heart Rate Variability (HRV)				95% CI		
Randomization	Mean RMSSD (ms)^*^	Standard Error	Mean Difference	Lower	Upper	P-Value
**Pre-CRI:SIS**						
**Control**	39.0	3.96	0.67	−10.68	12.03	0.91
**Intervention**	38.3	4.11				
**Post-CRI:SIS**						
**Control**	31.8	2.91	−11.55	−19.90	−3.20	0.007
**Intervention**	43.3	3.03				
**State Trait Anxiety Index (STAI)**				**95% CI**		
**Randomization**	**Mean Score**	**Standard Error**	**Mean Difference**	**Lower**	**Upper**	**P-Value**
**Pre-CRI:SIS**						
**Control**	34.3	1.03	−2.57	−5.52	0.38	0.09
**Intervention**	36.9	1.04				
**Post-CRI:SIS**						
**Control**	34.2	1.08	−1.64	−4.58	1.30	0.27
**Intervention**	35.8	1.07				

*Least Square Means (LSMEANS) of RMSSD; Root Mean Square Standard Deviation. An increase in RMSSD correlates with an increase in HRV and decrease in physiologic stress. LSMEANS are averages of two Pre-CRI:SIS and two Post-CRI:SIS shifts adjusted for number of steps taken.

#### STAI.

We received 317 (97.8%) post-shift survey responses across our 324 clinical shift data collections among 81 participants (41 control and 40 intervention). Pre-CRI:SIS, the reported mean SA score of 34.3 points (SE, 1.0) for the control group, and a mean SA score of 36.9 points (SE, 1.1) for the intervention group. Post-CRI:SIS, the reported mean SA score was 34.2 points (SE, 1.0) for the control group, and 35.8 points (SE 1.1) for the intervention group. Pre-CRI:SIS, there was no significant mean difference between the control and the intervention groups (−2.57 points, 95% CI, −5.52 to 0.38; **P* *= 0.09). Post-CRI:SIS, there was no significant mean difference between the groups (−1.64 points, 95% CI, −4.58 to 1.30; **P* *= 0.27).

## Discussion

To our knowledge, this is the first randomized control trial to evaluate the use of a rapidly adaptive simulation intervention on physiologic measures of stress among emergency physicians caring for acutely ill patients. This trial demonstrated the effectiveness of an adaptive COVID simulation intervention (CRI:SIS) on physician stress as measured by HRV during clinical shifts and compared this approach to clinicians who received no simulation intervention. The findings indicate a significant increase in HRV occurred in clinical shifts immediately following the completion of CRI:SIS in the intervention group compared to the control group as measured by RMSSD, correlating with increased parasympathetic tone (decreased stress) for the participants that received the intervention. To our knowledge, we are the first prospective clinical trial to demonstrate efficacy of a simulation intervention on physiologic stress in emergency physicians. In a systematic review of HRV as a measure of stress, [35] of the 16 articles reviewed, only one study used a randomized control trial experimental design but failed to demonstrate significant improvement in stress. [43–45] We believe these findings could be instrumental in reducing stress and burnout among physicians, particularly in the setting of a future pandemic, disasters, or even more chronically observed surges in healthcare worker stress from hospital crowding [12,43,46–49]

Our findings that CRI:SIS as a simulation intervention was able to decrease levels of stress in physician participants may reflect an increased level of confidence in the intervention group. During debriefing, participants had the opportunity to reflect on their own clinical experiences related to COVID-19 care. The debriefer for every intervention session was not only a simulation faculty member trained in debriefing skills, but also an attending physician in emergency medicine with the shared experience of caring for acutely ill patients with COVID-19. The intervention group had the opportunity to reflect on their performance in terms of what went well and what could be improved, receiving constructive feedback highlighting both positive actions and areas for improvement in a safe and supportive environment by a seasoned clinician. Participants also had the opportunity to process any emotional reactions they experienced during the simulation. These responses were most notable for the scenarios involving managing vaccine hesitancy and transitioning a patient to comfort measures only (CMO). By actively engaging in the CRIS:IS debriefing sessions, participants may have been able to leverage the experience gained from the simulations to build competence, resilience, and confidence in their abilities. While specific references on the direct impact of simulation debriefing on confidence may be limited, there are numerous studies that highlight the effectiveness of simulation-based training in enhancing learning outcomes and performance improvement. [50–52]

We did not find a statistically significant difference in the State-Trait Anxiety Index between the control and intervention groups. Trait anxiety refers to a trait or personality. In contrast, state anxiety reflects the psychological and physiological transient reactions directly related to adverse situations in a specific moment. While our HRV results indicate that a simulation intervention mitigates physiologic stress, we did not observe a difference in the STAI survey results. Akbar et al. reported similar results in their study of physician stress during electronic health record in-box work. [53] Several reasons may explain these findings. Firstly, STAI results were derived from self-report of stress and anxiety by participants. One of the main limitations of previous studies measuring stress is the reliance on self-reported measures of well-being. [54,55] In addition to not directly measuring stress per se, self-report approaches have several limitations for stress monitoring in the workplace. When people subjectively report how they feel, their evaluation could be affected by memory bias and emotion recognition, regulation, and expression biases. [56–59] Administering surveys for self-reports at the end of a clinical shift may also be less accurate since they require the full cognitive attention of the participant at a time when they may be eager to leave the hospital. Qualitative interviews with emergency physicians described sentiments of pride and responsibility to “rise up” to the challenge during the pandemic as a professional obligation and duty to their patients, which may potentially mask or bias their perceptions of stress or anxiety. [6,60,61] Other explanations may include physicians’ ability to mount physiological adaptations (e.g., improved autonomic regulation) or habituation of physiological responses that may occur independently of anxiety reduction. [62] Finally, since HRV reflects physiologic responses to stress, it may be able to detect more subtle or earlier signs of stress response or anxiety. This suggests that our intervention may be useful in mitigating negative effects on clinicians before it rises to the level of conscious awareness in our participants.

## Limitations

Our study included some limitations. This study was conducted at two hospitals at a single site, and the study only enrolled physicians and no other members of the health care team. Although physicians are an important target group for measuring stress in the setting of a pandemic, it may limit the generalizability of our findings. Future studies should consider recruiting participants at multiple sites with a more diverse sample population including other clinicians and nurses. Due to the nature of the simulation intervention, participants were not blinded to the study. We feel this is not a limitation because while lack of blinding could impact the self-reported STAI results post-CRI:SIS, we do not believe this impacted the post-CRI:SIS physiologic data collected in the intervention vs. control group. A lack of detectable difference in STAI results may be a potential limitation in this study’s ability to demonstrate differences in self-reported anxiety due to our intervention. We were unable to control for the number of COVID-19 patients and resuscitations evaluated during individual clinical shifts. However, based on our post-shift survey results, we determined there was no significant difference in the number of resuscitations between the control and intervention groups. The control arm did not receive any educational interactions or interactions with the instructional team; further studies may be needed to determine whether the observed effects were attributable to the simulation format itself or to other elements such as structured human interaction. Future work may also include measurements of clinical patient outcomes to determine potential impact on care quality and delivery as it relates to changes in clinician stress. Additional biomarkers of stress (e.g., blood glucose levels, hormones such as cortisol and epinephrine, body temperature, electrodermal activity) can also be measured to correlate with HRV results. [63] Finally, the effects of healthcare simulation on other domains of stress beyond the physiological, including emotional, cognitive, and social dimensions, may be explored to characterize its impact on overall stress in physicians. [64]

## Conclusions

Our findings indicate that emergency physicians who participated in a rapidly adaptive simulation intervention targeting COVID-19 care, CRI:SIS, demonstrated reduced physiologic stress as measured by increased heart rate variability. This suggests that an adaptive simulation technology focusing on quality and safety for a serious public health crisis may be a unique approach to concurrently prevent burnout and improve evidence-based knowledge translation for front-line emergency physicians. Given the stress on our emergency care system and its challenges in keeping up with the rising demand for acute care needs, healthcare administrators and policymakers will need to prioritize emergency physician well-being to ensure the highest quality patient care in the future.

## Supporting information

S1 Table Final Iteration of Intervention Scenarios. Summary of the intervention scenarios used in the study, detailing their design, objectives, and critical actions.
(DOCX)

S2 Table Post-Clinical Shift Survey. (DOCX)

S1 Fig Timeline of CRI:SIS adaptations in response to changes in best practices during the COVID-19 pandemic. An illustrative overview of key COVID-19 pandemic developments, including changes in Yale-New Haven Hospital COVID-19 Emergency Department Task Force protocols and clinical scenarios. Selected protocol updates (top) and simulation scenario modifications (bottom) are examples of the rapidly adaptive nature of CRI:SIS over the course of the clinical trial. Key events (flags) provide a national context for these changes.
(DOCX)

S1 checklist CRISIS CONSORT Checklist. (PDF)
