# Supplementary material for: An adaptive simulation intervention decreases emergency physician physiologic stress while caring for patients during COVID-19: A randomized clinical trial
Source: PLoS One. 2025 Sep 3;20(9):e0331488. doi: 10.1371/journal.pone.0331488 (PMC12407420; doi:10.1371/journal.pone.0331488)
Supplement: S1 Table — Summary of the intervention scenarios used in the study, detailing their design, objectives, and critical actions. (DOCX) [file pone.0331488.s001.docx]

**S1 Table. Final Iteration of Intervention Scenarios.** Summary of the intervention scenarios used in the study, detailing their design, objectives, and critical actions.

| **Scenario** | **Objectives** | **Critical Actions** |
| --- | --- | --- |
| Scenario 1: Severe COVID-19 with emphasis on goals of care  A 92-year-old woman with multiple comorbidities and severe COVID-19 | - Presentation immediately suggests a high risk of death necessitating goals of care discussion - Highlights hospital policy surrounding physicians conferring Do Not Resuscitate (DNR) and Do Not Intubate (DNI) orders | - Engage in goals of care discussion with a family member to determine the treatment pathway - Utilize hospital order sets for facilitating Comfort Measures Only (CMO) status - Utilize the hospital pathway for a severely ill COVID-19 patient (dependent on goals of care discussion) |
| Scenario 2: Mild COVID-19  A 73-year-old man with fever, respiratory rate (RR) <25, and spO2 above 94% with exertion | - Recognize that the patient’s severity of illness does not necessitate hospital admission - Discharge from the emergency department (ED) with close follow-up | - Order an outpatient COVID test - Contact the patient’s primary care physician - Discuss the patient’s expected course with return precautions - Address vaccine hesitancy |
| Scenario 3: Moderate to severe COVID-19  A 58-year-old patient presenting with hypoxia requiring hospital admission and deterioration in ED requiring a higher level of care | - Understand stratification of patients who are stable for floor admission vs. higher level of care - Gain skill in using the Quick COVID-19 Severity Index (CQSI)[^22^](https://web.endnote.com/citations/eyJkaXNwbGF5VGV4dCI6IjIyIiwiY2l0YXRpb25zIjpbeyJncm91cEd1aWRzIjpbIjk4YjY2OGFkLWRiZDItNGM1OS1iZTQzLTI3MTE5MWFkZDQwMiJdLCJndWlkIjoiNmMwZGI3NmQtMDA5Yy00NmE0LWI3ZjktN2MzMDgzODA0NTVkIiwiYmlibGlvQ29udGVudCI6W3sicGFnZXMiOiI0NDItNDUzIiwiZGF0ZSI6Ik9jdCIsImxhbmd1YWdlIjoiZW5nIiwiZWxlY3Ryb25pY1Jlc291cmNlTnVtYmVyIjoiMTAuMTAxNi9qLmFubmVtZXJnbWVkLjIwMjAuMDcuMDIyIiwia2V5d29yZHMiOlsiQWRvbGVzY2VudCIsIkFkdWx0IiwiQWdlZCIsIkJldGFjb3JvbmF2aXJ1cyIsIkNvdmlkLTE5IiwiQ09WSUQtMTkgVGVzdGluZyIsIkNsaW5pY2FsIExhYm9yYXRvcnkgVGVjaG5pcXVlcyIsIkNvcm9uYXZpcnVzIEluZmVjdGlvbnMvKmNvbXBsaWNhdGlvbnMvKmRpYWdub3Npcy90aGVyYXB5IiwiKkVtZXJnZW5jeSBTZXJ2aWNlLCBIb3NwaXRhbCIsIkZlbWFsZSIsIkh1bWFucyIsIk1hbGUiLCJNaWRkbGUgQWdlZCIsIk94eWdlbiBJbmhhbGF0aW9uIFRoZXJhcHkiLCJQYW5kZW1pY3MiLCJQbmV1bW9uaWEsIFZpcmFsLypjb21wbGljYXRpb25zLypkaWFnbm9zaXMvdGhlcmFweSIsIlJlc3BpcmF0b3J5IEluc3VmZmljaWVuY3kvdGhlcmFweS8qdmlyb2xvZ3kiLCJSZXRyb3NwZWN0aXZlIFN0dWRpZXMiLCJSaXNrIEFzc2Vzc21lbnQvbWV0aG9kcyIsIlNBUlMtQ29WLTIiLCIqU2V2ZXJpdHkgb2YgSWxsbmVzcyBJbmRleCIsIllvdW5nIEFkdWx0Il0sInZvbHVtZSI6Ijc2IiwiZ3VpZCI6IjZjMGRiNzZkLTAwOWMtNDZhNC1iN2Y5LTdjMzA4MzgwNDU1ZCIsInJlZmVyZW5jZVR5cGUiOiIxNyIsInNlY29uZGFyeVRpdGxlIjoiQW5uIEVtZXJnIE1lZCIsIm51bWJlciI6IjQiLCJpc2JuIjoiMDE5Ni0wNjQ0IChQcmludClccjAxOTYtMDY0NCIsIm5vdGVzIjoiMTA5Ny02NzYwXHJIYWltb3ZpY2gsIEFkcmlhbiBEXHJSYXZpbmRyYSwgTmVhbCBHXHJTdG95dGNoZXYsIFN0b3l0Y2hvXHJZb3VuZywgSCBQYXRyaWNrXHJXaWxzb24sIEZyYW5jaXMgUFxydmFuIERpamssIERhdmlkXHJTY2h1bHosIFdhZGUgTFxyVGF5bG9yLCBSIEFuZHJld1xyUDMwIERLMDc5MzEwL0RLL05JRERLIE5JSCBISFMvVW5pdGVkIFN0YXRlc1xyUjAxIERLMTEzMTkxL0RLL05JRERLIE5JSCBISFMvVW5pdGVkIFN0YXRlc1xyVUwxIFRSMDAxODYzL1RSL05DQVRTIE5JSCBISFMvVW5pdGVkIFN0YXRlc1xySm91cm5hbCBBcnRpY2xlXHJPYnNlcnZhdGlvbmFsIFN0dWR5XHJSZXNlYXJjaCBTdXBwb3J0LCBOLkkuSC4sIEV4dHJhbXVyYWxcclJlc2VhcmNoIFN1cHBvcnQsIE5vbi1VLlMuIEdvdid0XHJWYWxpZGF0aW9uIFN0dWR5XHJVbml0ZWQgU3RhdGVzXHIyMDIwLzEwLzA2XHJBbm4gRW1lcmcgTWVkLiAyMDIwIE9jdDs3Nig0KTo0NDItNDUzLiBkb2k6IDEwLjEwMTYvai5hbm5lbWVyZ21lZC4yMDIwLjA3LjAyMi4gRXB1YiAyMDIwIEp1bCAyMS4iLCJhY2Nlc3Npb25OdW1iZXIiOiIzMzAxMjM3OCIsImFic3RyYWN0IjoiU1RVRFkgT0JKRUNUSVZFOi) - Understand strategies for improving oxygenation | - Admission with appropriate therapeutics (Remdesivir, steroids, aspirin) - Utilize the Quick COVID-19 Severity Index (QCSI) & arterial measurement of PaO2/FiO2 (P/F) ratio for risk stratification - Use modalities to improve oxygenation - proning, high flow oxygen, BiPAP |
| Scenario 4: Severe COVID-19 requiring emergency intubation  A 50-year-old patient requiring immediate intubation. Ventilator management leads to barotrauma | - Understand the management of a difficult airway in a patient with minimal oxygen reserve - Practice ventilator management using ARDS Net - Discuss barotrauma injury in high PEEP | - Use a difficult airway algorithm - Manage increasing ventilator requirements with ARDS Net - Successfully diagnose and treat pneumothorax - Utilize hospital pathway for a severely ill COVID-19 patient |
