# Supplementary material for: An adaptive simulation intervention decreases emergency physician physiologic stress while caring for patients during COVID-19: A randomized clinical trial
Source: PLoS One. 2025 Sep 3;20(9):e0331488. doi: 10.1371/journal.pone.0331488 (PMC12407420; doi:10.1371/journal.pone.0331488)
Supplement: S2 Table — (DOCX) [file pone.0331488.s002.docx]

**S2 Table. Post-Clinical Shift Survey**

**1) Participant Number**

***A number of statements that people have used to describe themselves are given below. Read each statement and choose the response that best indicates how you feel in this moment as a result of your shift. There are no right or wrong answers. Do not spend too much time on any one statement but give the answer which seems to describe your present feelings best.***

**2) I feel calm**

- **Not At All**
- **Somewhat**
- **Moderately So**
- **Very Much So**

**3) I feel secure**

- **Not At All**
- **Somewhat**
- **Moderately So**
- **Very Much So**

**4) I am tense**

- **Not At All**
- **Somewhat**
- **Moderately So**
- **Very Much So**

**5) I feel strained**

- **Not At All**
- **Somewhat**
- **Moderately So**
- **Very Much So**

**6) I feel at ease**

- **Not At All**
- **Somewhat**
- **Moderately So**
- **Very Much So**

**7) I feel upset**

- **Not At All**
- **Somewhat**
- **Moderately So**
- **Very Much So**

**8) I am presently worrying over possible misfortunes**

- **Not At All**
- **Somewhat**
- **Moderately So**
- **Very Much So**

**9) I feel satisfied**

- **Not At All**
- **Somewhat**
- **Moderately So**
- **Very Much So**

**10) I feel frightened**

- **Not At All**
- **Somewhat**
- **Moderately So**
- **Very Much So**

**11) I feel comfortable**

- **Not At All**
- **Somewhat**
- **Moderately So**
- **Very Much So**

**12) I feel self-confident**

- **Not At All**
- **Somewhat**
- **Moderately So**
- **Very Much So**

**13) I feel nervous**

- **Not At All**
- **Somewhat**
- **Moderately So**
- **Very Much So**

**14) I am jittery**

- **Not At All**
- **Somewhat**
- **Moderately So**
- **Very Much So**

**15) I feel indecisive**

- **Not At All**
- **Somewhat**
- **Moderately So**
- **Very Much So**

**16) I am relaxed**

- **Not At All**
- **Somewhat**
- **Moderately So**
- **Very Much So**

**17) I feel content**

- **Not At All**
- **Somewhat**
- **Moderately So**
- **Very Much So**

**18) I am worried**

- **Not At All**
- **Somewhat**
- **Moderately So**
- **Very Much So**

**19) I feel confused**

- **Not At All**
- **Somewhat**
- **Moderately So**
- **Very Much So**

**20) I feel steady**

- **Not At All**
- **Somewhat**
- **Moderately So**
- **Very Much So**

**21) I feel pleasant**

- **Not At All**
- **Somewhat**
- **Moderately So**
- **Very Much So**

**22) How many resuscitations did you perform on this shift?**

**23) How many of those patients/resuscitations were known COVID+ or PUI?**

**What, if any, events from your shift stand out to you as having been particularly stressful?**
