# Supplementary material for: An adaptive simulation intervention decreases emergency physician physiologic stress while caring for patients during COVID-19: A randomized clinical trial
Source: PLoS One. 2025 Sep 3;20(9):e0331488. doi: 10.1371/journal.pone.0331488 (PMC12407420; doi:10.1371/journal.pone.0331488)
Supplement: S1 Fig — An illustrative overview of key COVID-19 pandemic developments, including changes in Yale-New Haven Hospital COVID-19 Emergency Department Task Force protocols and clinical scenarios. Selected protocol updates (top) and simulation scenario modifications (bottom) are examples of the rapidly adaptive nature of CRI:SIS over the course of the clinical trial. Key events (flags) provide a national context for these changes. (DOCX) [file pone.0331488.s003.docx]

**
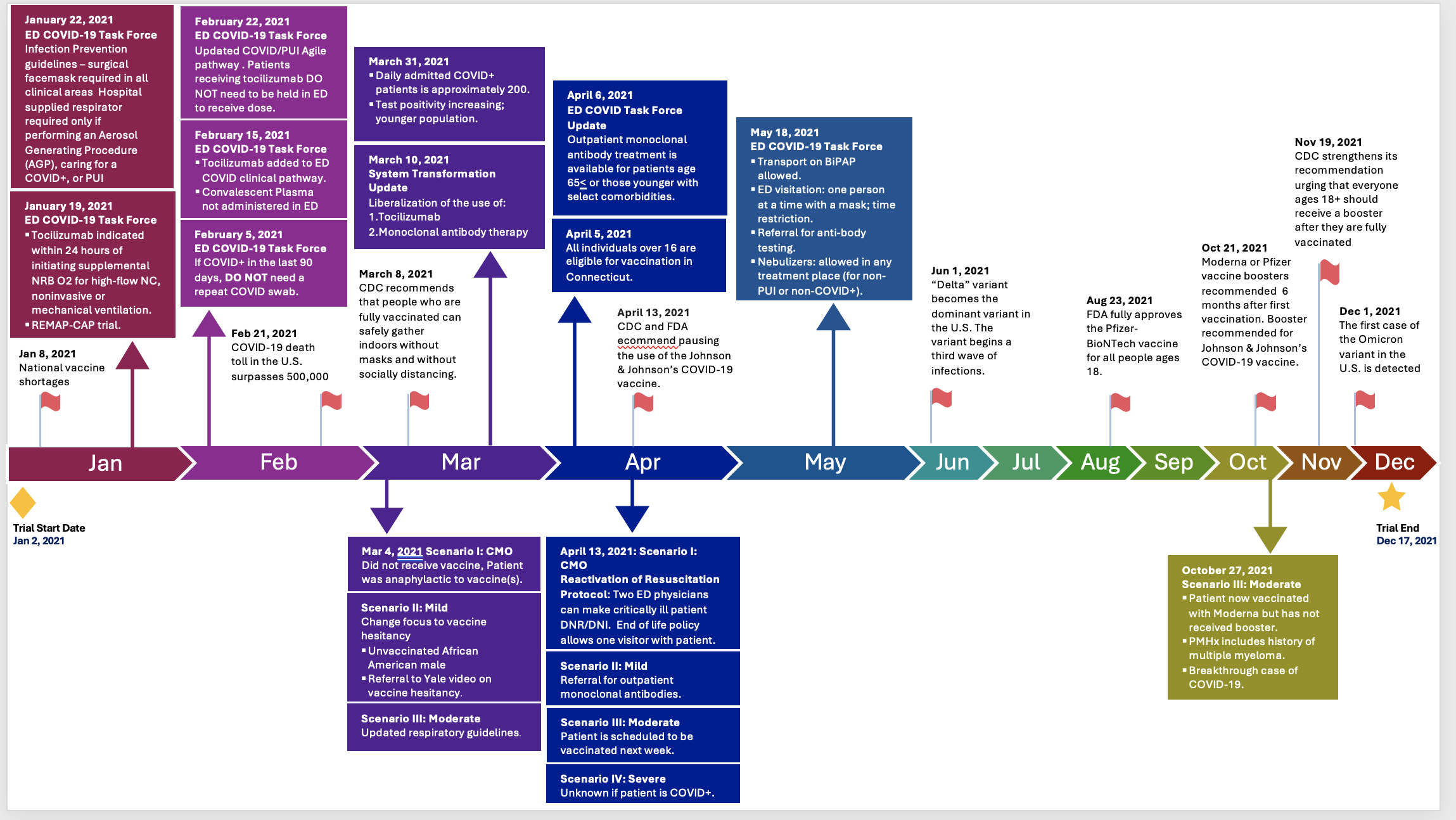
**

**S1 Fig. Timeline of CRI:SIS adaptations in response to changes in best practices during the COVID-19 pandemic.** An illustrative overview of key COVID-19 pandemic developments, including changes in YNHH COVID-19 ED Task Force protocols and clinical scenarios. Selected protocol updates (top) and simulation scenario modifications (bottom) are examples of the rapidly adaptive nature of CRI:SIS over the course of the clinical trial. Key events (flags) provide a national context for these changes.
